# Burrowing and unburrowing in submerged granular media through fluidization and shape-change

**DOI:** 10.3389/frobt.2025.1546407

**Published:** 2025-07-31

**Authors:** Aniruddha Nayak, Hoseung Seo, Nick Gravish, Michael T. Tolley

**Affiliations:** Department of Mechanical and Aerospace Engineering, University of California San Diego, La Jolla, CA, United States

**Keywords:** soft robot, burrowing, granular media, shape change, fluidization, untethered

## Abstract

Subterranean exploration in submerged granular media (GM) presents significant challenges for robotic systems due to high drag forces and the complex physics of GM. This paper introduces a robotic system that combines water-jet-based fluidization for self-burrowing in submerged environments and an untethered, volume-change mechanism for burrowing out. The water-based fluidization approach significantly reduces drag on the robot, allowing it to burrow into GM with minimal force. To burrow out, the robot uses a soft, inflatable bladder that undergoes periodic radial expansion, inspired by natural systems such as razor clams. Experimental results demonstrate that increased water flow rates accelerate the burrowing process, while the unburrowing mechanism is effective at varying depths. Comparisons between pneumatic and hydraulic untethered systems highlight trade-offs in terms of operational time and unburrowing speed. This work advances the capabilities of robots in underwater environments, with potential applications in environmental monitoring and underwater archaeology.

## 1 Introduction

Exploration of granular media (GM) submerged in water is critical for applications in marine research, environmental monitoring, and underwater archaeology (Cui et al., 2023; Dunbabin and Marques, 2012). Furthermore, advancement of robots for exploration of soils can improve understanding and inspection of geotechnical systems (Martinez et al., 2021). Traditional robotic systems designed for locomotion on land, in air, and in water face considerable challenges when operating in granular environments. Within granular media an intruder will experience large drag forces that can result in solid-like or liquid-like flow of the grains that can challenge robot motion (Gravish et al., 2010; Ding et al., 2011). These issues necessitate new approaches to robot design and control mechanisms.

Previous biological burrowers, including self-burying seeds (Evangelista et al., 2011), tree roots (Clark et al., 2003), worms (Dorgan, 2015), mole crabs (Trueman, 1970), sandfish (Macdonald, 2015), lizards (Maladen et al., 2009), octopuses (Montana et al., 2015) and razor clams (Trueman, 1967), employ various strategies to create anisotropic forces necessary for burrowing in GM, such as tip extension, cyclic expansion, asymmetric gaits, and localized fluidization via fluid flow. Inspired by these organisms, previous studies have explored mechanisms for burrowing in GM, employing anisotropic appendages (Chopra et al., 2023; Drotman et al., 2022; Treers et al., 2022; Kim et al., 2023; Zhang et al., 2024), tip growth (Sadeghi et al., 2017; Gyeol et al., 2023), expansion segments (Ortiz et al., 2019; Omori et al., 2009; Naziri et al., 2024; Weiwei et al., 2019), screw mechanism (Bagheri et al., 2023), undulatory motion (Maladen et al., 2011), or the use of air jetting to locally fluidize GM to lower drag and facilitate motion (Naclerio et al., 2021). However, these studies focused primarily on dry GM, whereas in submerged conditions, GM and fluid flow exhibit different mechanical interactions.

Although recent advances have explored burrowing methods for submerged GM, such as employing cyclic axial expansion inspired by bivalve species (e.g., razor clams) (Winter et al., 2014; Huang and Tao, 2022; Germann and Carbajal, 2013) and developing underwater drilling robots based on earthworms (Isaka et al., 2019), these existing systems are limited by their reliance on tethered setups or their inability to burrow both in and out of submerged GM.

On the other hand, bioinspired unburrowing (i.e., burrowing out of GM) robots have been less studied, except for a few examples such as a previous work that utilizes cyclic body expansions inspired by razor clams (Tao et al., 2020), and there is a notable gap in systems capable of achieving both burrowing and unburrowing in submerged GM.

To address these limitations, this paper presents a robotic system capable of both burrowing in and out of shallow submerged GM. Our contributions are twofold: first, we introduce a water-jet-based fluidization technique that enables the robot to burrow into submerged GM with minimal drag; second, we propose an untethered, soft, volume-change mechanism that allows the same robot to burrow out of GM. We provide experimental validation of both techniques, demonstrating their effectiveness in reducing drag forces and enabling autonomous operation in submerged environments.

The remainder of this paper is structured as follows: Section 2 details the design and operation of our robotic system. In Section 3 we go over the experimental results, followed by demonstration of untethered robotic implementation in Section 4. We conclude with a discussion of the system’s performance and potential applications and suggest directions for future work in Section 5.

## 2 Methods

### 2.1 Conceptual motivation and robot design

**FIGURE 1:**
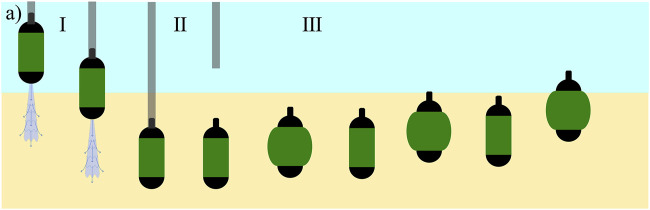
The burrowing and unburrowing mechanisms for the proposed system consisting of three phases: I) Self-burrowing in submerged GM using tethered fluidization. II) Tether detaches when the robot reaches the required depth. III) The robot self-burrows out of GM using periodic volume change.

The proposed robotic system employs two key mechanisms: 1) the use of water jet for local fluidization of submerged granular media (GM) for self-burrowing and 2) an untethered, radial expansion mechanism for burrowing out of submerged GM (Figure 1). By integrating these two features into a single system, the robot is capable of burrowing into submerged GM until the desired depth is reached, at which point the tethered fluidization system can be detached, allowing the robot to operate and to unburrow autonomously.

**FIGURE 2:**
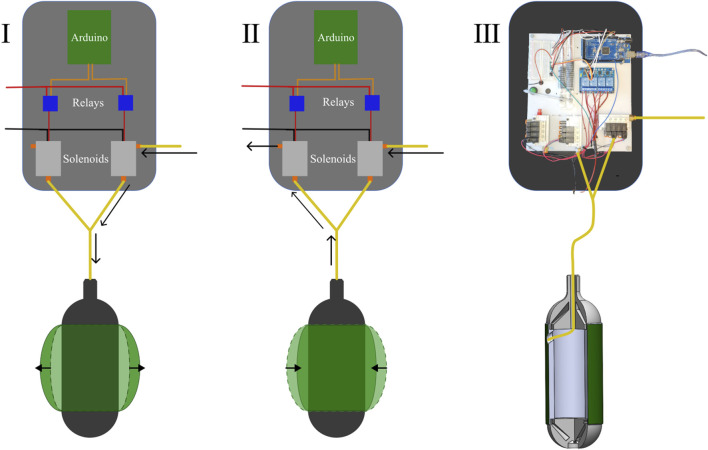
I) The pathway of air during the supply cycle. (The black arrows show the pathway the air takes) II) The pathway of air during the exhaust cycle. (The black arrows show the pathway the air takes) III) The physical pneumatic control board and the connection schematic for the bladder.

Our unburrowing mechanism relies on the periodic radial expansion of a soft bladder fabricated with thermoplastic polyurethane (TPU), which, through mechanical interactions with the surrounding GM, generates enough force to push the robot upward. The bladder, shaped like a toroid, was made with TPU sheet of 0.12 mm thickness to resist damage from interactions with GM, and to withstand working pressures of 20–30 PSI; once fabricated, it was assembled over a 3D-printed body (Figure 2).

**FIGURE 3:**
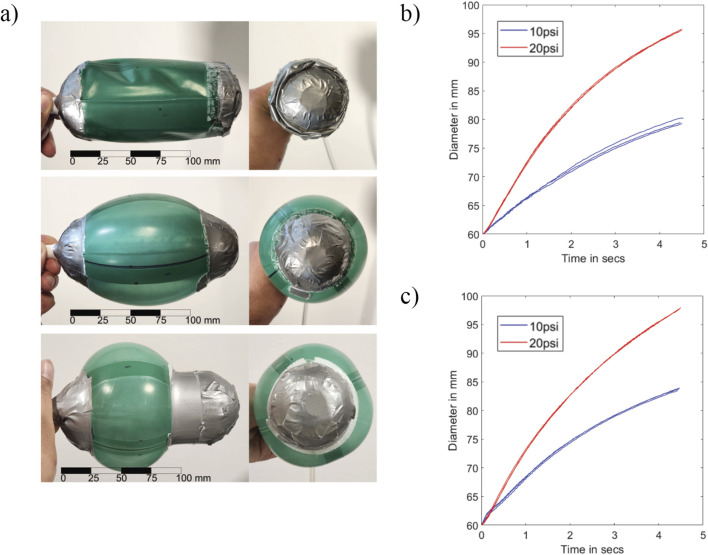
**(a)** Front and side views of the deflated full-skin variation, inflated full-skin variation bladder, inflated half-skin variation bladder (top to bottom). **(b)** Diameter vs. time for the full-skin bladder for 10 PSI and 20 PSI. **(c)** Diameter vs. time for the half-skin bladder for 10 PSI and 20 PSI. The x-axes for both plots are $T_{\mathrm{supply}}$ .

In order to explore how the shape of the bladder affects the unburrowing performance of the robot, we tested two variations of the bladder: full-skin and half-skin (Figure 3). The full-skin bladder covered the entire length of the center part of the robot, while the half-skin covered only half the length. Therefore, for the same maximum diameter of the bladder, the half-skin variation would require less fluid volume as compared to the full-skin version. This comparison allowed us to test the competing effects of the volume vs the rate of shape change, as we assumed that a smaller bladder could be inflated more quickly.

The radial expansion mechanism was initially controlled by an external control board consisting of a microcontroller (Arduino Mega), solenoid valves (SMC VQ110U) connected to pressure-regulated air supply, and relays (ELEGOO 4 Channel DC 5 V Relay Module); in later iterations, these components were housed within the robot body to achieve untethered operation. To regulate the extent of radial expansion, we controlled the time the bladder was allowed to expand $\left(T_{\mathrm{supply}}\right)$ , as well as the time the expanded bladder vented to the atmosphere $\left(T_{\mathrm{exhaust}}\right)$ . Therefore, the total control cycle time (*T_cycle_*) for one period of inflation and deflation of the bladder was given by the sum of *T_supply_* and *T_exhaust_* (Equation 1).

$$T_{\mathrm{cycle}}=T_{\mathrm{supply}}+T_{\mathrm{exhaust}}$$ (1)

### 2.2 Experimental setup for characterizing unburrowing behavior

We first performed unburrowing tests in a dry sand test bed to characterize the unburrowing behavior of the actuator. The reason for initial testing in dry GM, as opposed to submerged GM, is twofold. First, to ensure the repeatability of our data, we fluidized the test bed with air between each experiment to reset the volume fraction of the GM (Gravish et al., 2010). While this process is reliable and widely used for experiments in dry GM, resetting submerged GM is significantly more challenging due to interactions between the water and GM, and such methods are relatively underdeveloped. Second, since we used air to inflate the TPU bladder, we were concerned that the buoyancy effect may influence the results of the experiments by aiding the unburrowing process; therefore, we first tested the mechanism in the worst-case scenario of dry test bed first, then in later demonstration showed that the mechanism also worked in submerged GM.

For the granular substrate, we used glass beads of 0.3 mm diameter to mimic the particle size of medium sand (0.2 mm–0.63 mm according to ISO 14688-1:2017). The dry sand test bed had dimensions of 420 mm × 420 mm wide, with a maximum sand depth of 350 mm, and was integrated with an air-based fluidization system to reset the GM to a uniformly packed state between experiments (Gravish et al., 2010). Before each experimental run, we placed our robot at the bottom of the sand bed while fluidizing the entire test bed. We then turned off the fluidizing bed, started the cyclic radial expansion of the bladder, and recorded the unburrowing performance of the robot from the bottom to the top of the sand bed.

To characterize the behavior and performance of the unburrowing mechanism, we performed a set of three trials with the half-skin variation. The robot in this configuration weighed 200 g. We regulated the supply pressure (connected to the fluidic control board) to 10 PSI. Due to the interactions with the surrounding GM, we assumed that the extent of radial expansion of the bladder at a given internal pressure would be inversely proportional to the depth of the sand in the robot. To track the position of the robot, we attached a long, hollow aluminum tube with a diameter 6 mm to the top of the robot to protrude out of the sand throughout the experiment in order to track the position of the robot as it unburrowed (Figure 5a). After each test, we used a video analysis software (Tracker Version 6.1.6, Open Source Physics) to track the position of the robot over time.

### 2.3 Experimental setup for characterizing burrowing behavior

**FIGURE 4:**
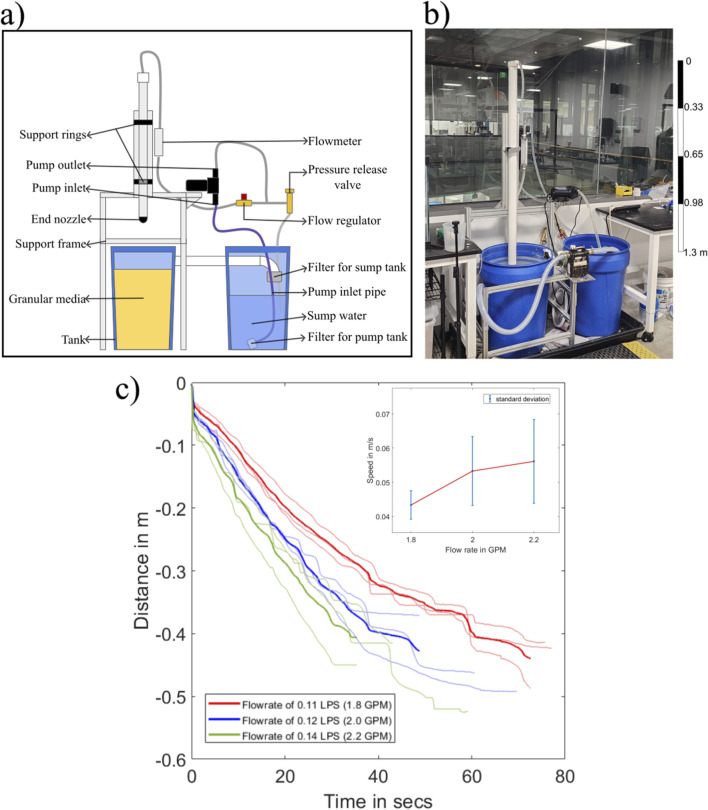
**(a)** Experimental setup to characterize the relation of self-burrowing rate vs. the flow rate of water. **(b)** The physical setup used to perform the self-burrowing experiments. **(c)** Recorded position of probe (in meters) over time (in seconds), which shows the effect of flow rate on the burrowing speed.

We evaluated the effectiveness of our water jet-based fluidization mechanism for burrowing in submerged GM by measuring the self-burrowing rate of an intrusion probe in a test bed (cylinder with diameter 500 mm and maximum submerged GM depth of 550 mm) filled with the same granular substrate used for dry test bed, now submerged in water (Figure 4). The probe consisted of a PVC tube of diameter 60 mm with a nozzle on the bottom end and was connected to a water supply on the top. Two hoops constrained the PVC tube to only allow vertical motion. We used a diaphragm pump to generate flow rates of water around a nominal operating flow of 0.12 L/s (liters per second), which were measured using an inline flow meter. After setting the flow to a specific value we released the probe, which then burrowed into the submerged GM due to its own weight and the fluidizing effects of the water jet. We repeated the test three times each at flow rates of 0.11 L/s , 0.12 L/s , and 0.14 L/s , and compared the burrowing speed of the probe.

As discussed previously, resetting the compaction ratio of submerged GM is more challenging than in dry GM; we initially observed that if the GM was not reset properly after each test, there were pockets of loosely packed GM in the region where the probe had burrowed into, leading to inconsistent results in the subsequent trials. In order to resolve this issue, we used an additional pump connected to a separate nozzle, which was used to stir up and fluidize the entire test bed. Afterward, the submerged GM was allowed to settle down, and we used a heavy plate to apply uniform pressure to the top surface of the GM. Using this process, we noticed increased consistency in the results.

## 3 Experimental results

### 3.1 Burrowing performance in submerged granular media (GM) with water-based fluidization

The results of the self-burrowing tests in submerged GM showed that when the flow rate increases, the rate at which the tube self-burrows increases (Figure 4c). We found that all three ranges of flow rate were able to self burrow to a depth of 0.4 m. However, the large variability in the results prevents us from identifying a clear trend in the data.

### 3.2 Unburrowing performance with the radial expansion mechanism

**FIGURE 5:**
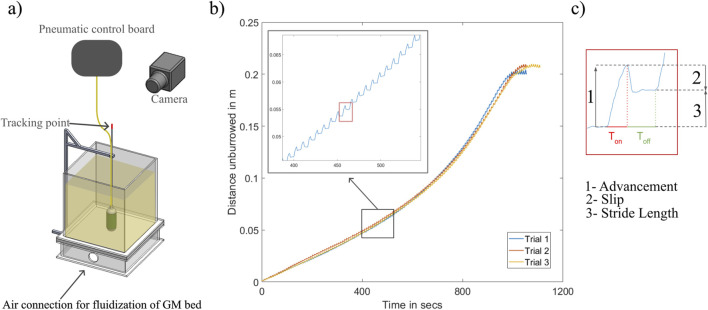
**(a)** Experimental setup used to measure burrowing-out data. **(b)** Distance burrowed out vs. Time. Distance=0 corresponds to the bottom of the test bed. Closeup of the plot shows locomotion pattern for each activation cycle. **(c)** Terminology used to understand the locomotion pattern.

For the unburrowing tests, we initially set $T_{\mathrm{supply}}$ to 3.5 s and $T_{\mathrm{exhaust}}$ to 4.5 s during each expansion-contraction cycle. The results of these tests showed that our developed mechanism exhibited similar locomotion properties as observed in the unburrowing gait of a razor clam (Figure 5b; Winter et al. (2014)). During the supply cycle the body is expanding and the robot advanced in the upward direction. We used the term Advancement for the distance burrowed out in the supply cycle. When the bladder was exhausting the body is shrinking and the robot slipped part of the way back in the downward direction. We use the term Slip for the distance burrowed in during the exhaust cycle. We refer to the net motion of the robot during an actuation cycle as the StrideLength (Equation 2).

StrideLength=Advancement−Slip (2)

**FIGURE 6:**
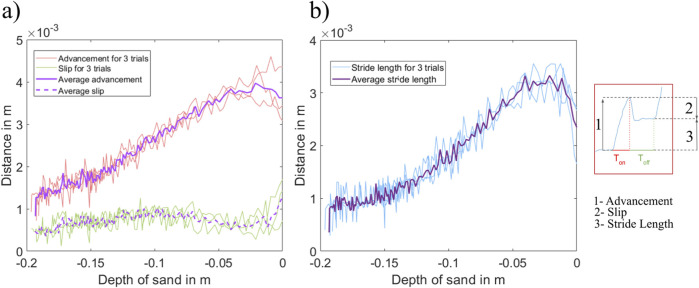
**(a)** Advancement and Slip vs. the depth of sand the robot is burrowed under. **(b)** StrideLength vs. the depth of sand the robot is burrowed under.

To evaluate the effect of the sand depth on these three components of the unburrowing cycle, we plotted the Advancement , Slip , and StrideLength of each cycle against the depth of GM (Figure 6). We observed that the Advancement for each cycle increased linearly with the reduction in the depth of sand. While the Slip for each cycle remained constant throughout most of the unburrowing process (Figure 6a).

### 3.3 Effect of cycle time on the unburrowing performance

Looking at the closeup of the locomotion pattern (Figure 5c), we observed that during the exhaust cycle, the Slip that the robot experienced occurred in the first 25% of the exhaust time $T_{\mathrm{exhaust}}$ . During the remaining time in $T_{\mathrm{exhaust}}$ , the robot stayed remained at a constant depth. We hypothesized that reducing the exhaust time in the cycle would reduce the total unburrowing time of the robot.

To test this hypothesis, we performed an additional set of tests, where we reduced $T_{\mathrm{exhaust}}$ from 4.5 s to 3.5 s, while $T_{\mathrm{supply}}$ remained unchanged at 3.5 s. We first plotted the depth of the robot vs. time in seconds. We normalized the y -axis data such that the robots reached depth 0 (surface of the sand) at the end of the unburrowing experiment. Since the robots had a slight variation in the starting depth across trials, we only compared the data starting at a common depth of 180 mm.

**FIGURE 7:**
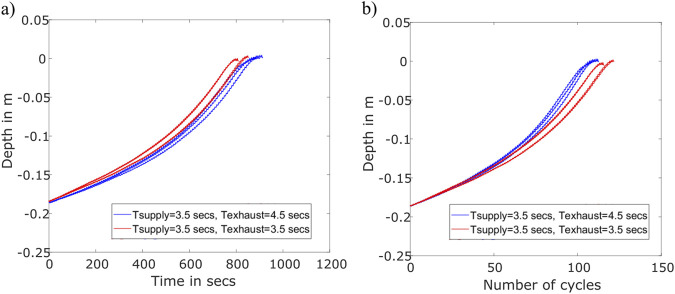
Measuring the effect of bladder deflation time on unburrowing speed and efficiency. **(a)** Depth unburrowed (in meters) vs. time required (in seconds). **(b)** Depth unburrowed (in meters) vs. Number of cycles required.

The results showed that reducing the exhaust time decreased the unburrowing time. The robot with the shorter cycle time (7 s) reached the surface after an average time of 780 s compared to 890 s for the original cycle time (8 s; Figure 7a), which corresponds to a 12.5% improvement in the unburrowing time. However, looking at depth vs number of cycles, we also plotted the position of the robot against the number of cycles the bladder has expanded and contracted. Since the total cycle time (i.e., $T_{\mathrm{supply}}+T_{\mathrm{exhaust}}$ was 8 s for the original gait and 7 s for the modified gait) this analysis showed that the robot burrows out in almost 110 cycles for both gaits (Figure 7b). This analysis elaborated an important trade-off for an untethered system: In the case where unburrowing speed were paramount then a shorter cycle time would be better (where the bladder may not be fully deflated each cycle), however if a smaller number of total cycles were preferred (possibly for energy efficiency, or due to a limited supply of gas for inflation) then a slightly longer cycle time may be preferred.

### 3.4 Effects of the varying weight of the robot and the shape of the bladder on the unburrowing performance

To understand how the unburrowing performance of the robot was affected by the shape of the bladder and the intrinsic weight of the robot, we performed additional unburrowing experiments in the dry GM test bed. For each configuration, we conducted three trials each with varying $T_{\mathrm{supply}}$ at 2, 3, 3.5, and 4 s, while $T_{\mathrm{exhaust}}$ was kept at $T_{\mathrm{supply}}+1$ seconds. Since the air supply pressure was kept constant as in previous tests at 10 psi, a larger $T_{\mathrm{supply}}$ corresponds to larger maximum diameter of the bladder during each cycle. After each experiment, we plotted the average time to unburrow against $T_{\mathrm{supply}}$ for both bladder variations.

**FIGURE 8:**
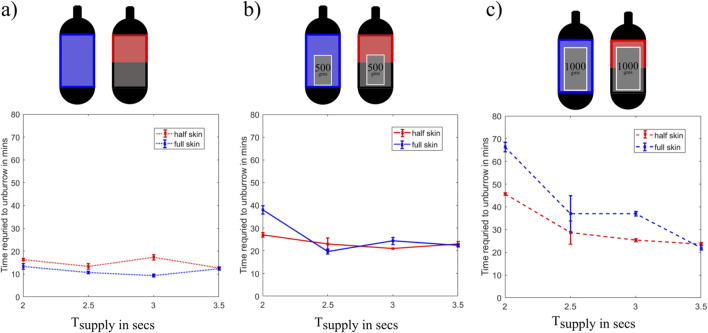
**(a)** Time required to unburrow in minutes vs. Supply time in seconds for a robot just burrowing out without any extra weight. **(b)** Time required to unburrow in minutes vs. Supply time in seconds for a robot burrowing out with an extra 500-g weight in the internal cavity. **(c)** Time required to unburrow in minutes vs. Supply time in seconds for a robot burrowing out with an extra 1,000-g weight in the internal cavity.

The first set of experiments was performed without adding extra weight to the robot, which, in this configuration weighed 200 g. We observed that the full-skin variation, on average, unburrowed 21% faster than the half-skin variation (Figure 8a). For both variations, the unburrowing performance remained consistent for all $T_{\mathrm{supply}}$ tested.

To observe how the weight of the robot affects its burrowing-out performance, we then conducted the same tests after adding a 500-g weight to the internal cavity of the 3D-printed core of the robot (i.e., total weight was 700 g). In this configuration, the half-skin variation burrowed out 29% faster than the full-skin variation for $T_{\mathrm{supply}}$ of 2 s (Figure 8). For all other $T_{\mathrm{supply}}$ tested, both variation exhibited similar unburrowing performance.

Finally, we also conducted experiments after adding a 1,000-g weight to the robot, which increased the total weight to 1,200 g. For this configuration, we observed that the half-skin variation burrowed-out faster than the whole-skin variation for most of the $T_{\mathrm{supply}}$ tested; the half-skin variation was, on average, 31%, 22.4%, and 32.4% faster than the full-skin version at $T_{\mathrm{supply}}$ of 2, 2.5, and 3 s, respectively (Figure 8c). We also noticed that for both bladder variations, the unburrowing performance of the robot increased significantly with increase in $T_{\mathrm{supply}}$ ; the larger the bladder was allowed to expand in each cycle, the faster the robot burrowed out. Ultimately this experiment allowed us to select an overall $T_{\mathrm{supply}}$ time of 3.5 s because the unburrowing behavior was only weakly influenced by the added weight across the three experiments.

## 4 Demonstration of a robot capable of burrowing using water jet and untethered self-unburrowing using cyclical radial expansion

So far, we have characterized the two main parts of our proposed method—burrowing using water jet-based fluidization and unburrowing using cyclical radial expansion of the bladder. In this section, we design and test a robot that combines both of these functionalities, using a tether to burrow into submerged GM, detaching, and burrowing out untethered. We examined two approaches to achieve untethered unburrowing: embedding a CO_2_ canister to supply gas for shape change, and using a gear pump and an internal liquid bladder.

### 4.1 Burrowing out using an onboard gas supply from a CO_2_ canister

#### 4.1.1 Design and fabrication

**FIGURE 9:**
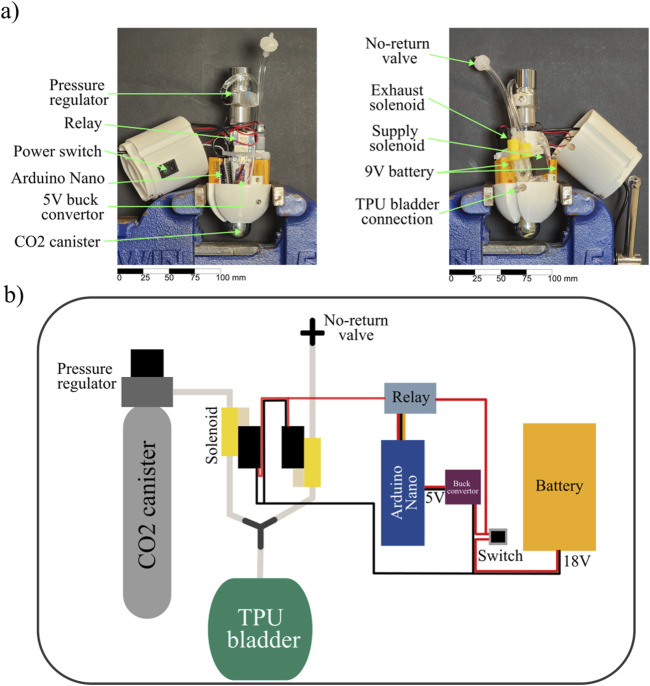
Design of an untethered shape change unburrowing system with an onboard supply of ${\text{CO}}_{2}$ . **(a)** Photographs of the internal structure of the robot, with components of the electro-pneumatic system labeled. **(b)** The schematic of the electro-pneumatic system onboard, with components labeled.

We designed a pneumatic system using a pressurized 45 g CO_2_ canister as our air supply (Figure 9a). We regulated the pressure from the ${\text{CO}}_{2}$ canister using a compact pressure regulator. We observed that even if we set the pressure at 10 PSI when a new canister was installed, the pressure was not constant throughout the entire canister life. Towards the end of the capacity of the canister, the pressure would be higher than the pre-set 10 PSI. To achieve untethered operation, we first 3D-printed the pill-shaped hollow body of the robot, which had a diameter of 74 mm, using polylactic acid (PLA). We then embedded two 18 V 3/2 solenoid valves (SMC VQ110U) to control the flow to and from the TPU bladder, a relay (Adafruit STEMMA Non-Latching Mini Relay) to control the solenoids, two 9 V batteries in series as power source, a microcontroller (Arduino Nano), and a buck converter (Adafruit MPM3610 5 V Buck Converter) to power the microcontroller inside the cavity of the robot (Figure 9b). These components serve similar functionalities as those in the pneumatic control board used in the tethered experiments, however, we chose them to mimimize the total volume of the control system. The cyclic inflation/deflation of the bladder was initiated by a switch installed on the outer wall of the robot.

#### 4.1.2 Operation and design limitations

**FIGURE 10:**
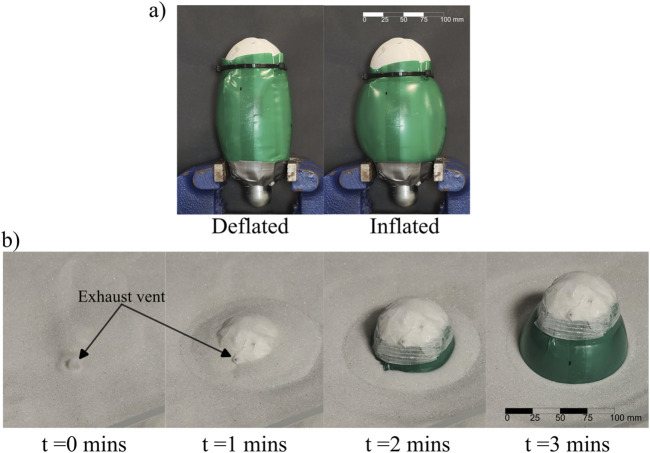
Experimental testing of the ${\text{CO}}_{2}$ powered unburrowing robot in dry GM from a shallow depth. **(a)** Photographs of the untethered robot in the deflated (left) and the inflated (right) state. **(b)** Photographs of the robot unburrowing at discrete time intervals. The robot was turned on at t=0 min.

For the untethered unburrowing tests using ${\text{CO}}_{2}$ canister, we set $T_{\mathrm{supply}}$ to 2.5 s and $T_{\mathrm{exhaust}}$ to 5.0 s (Figure 10a). We kept the $T_{\mathrm{supply}}$ slightly lower than in our previous tests to compensate for the increase in the pressure over the life of the canister. We also set $T_{\mathrm{exhaust}}$ as twice as large as $T_{\mathrm{supply}}$ to ensure that the robot had enough time to vent the bladder through the small opening on the top of the robot. With this gait, one canister, on average, lasted 480 s of operation, which corresponds to approximately 64 cycles.

We tested the ability of the ${\text{CO}}_{2}$ powered robot to unburrow in dry GM (Figure 10). During the experiments, we found out that 64 cycles of the expansion of the bladder were only enough to locomote 0.25 body lengths under the surface of the GM. Thus with the ${\text{CO}}_{2}$ energy source the robot could not fully unburrow from even shallow depths. The reason for this is the limited energy density of the ${\text{CO}}_{2}$ canister for this untethered pneumatic pressure-based implementation. If burrowed just under the GM, the robot took 3 min to unburrow a quarter of its body (Figure 10b).

### 4.2 Water pump-based hydraulic untethered unburrowing approach

In order to overcome the limitations of the ${\text{CO}}_{2}$ canister-based approach where the robot did not have a sufficiently large operation time, we also designed and tested a closed-loop hydraulic system for untethered unburrowing.

#### 4.2.1 Design and fabrication

**FIGURE 11:**
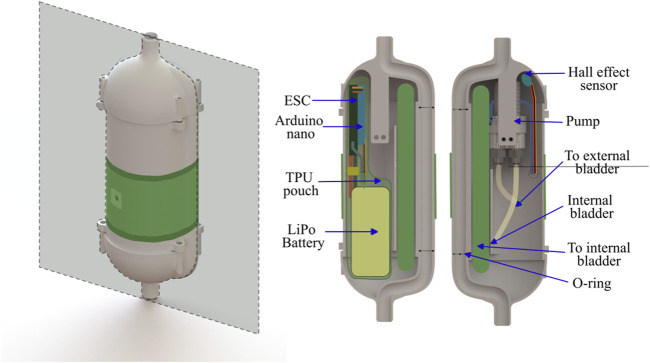
3D CAD rendering of the water robot with shape change actuated by a water pump, with the cross-section plane (Left). The cross-section of the inside of the robot with primary components labeled (Right).

In this final approach, we constructed a robot that was 21.5 cm in length and 7 cm in diameter that is capable of fluidization burrowing and untethered unburrowing. Instead of using a compressed ${\text{CO}}_{2}$ canister we implemented a pump to cycle water from an internal bladder to an external bladder during $T_{\mathrm{supply}}$ and *vice versa* during $T_{\mathrm{exhaust}}$ for cyclic radial expansion of the external bladder (Figure 11. The embedded system consisted of a DC gear pump (TCS MGD1000F-PK-V-EQi High Flow), a microcontroller (Arduino Nano), a drone electronic speed controller (Readytosky Bidirectional 20 A Brushless ESC) to control the gear pump, and a lithium polymer battery (14.8 V, 1,300 mAh).

**FIGURE 12:**
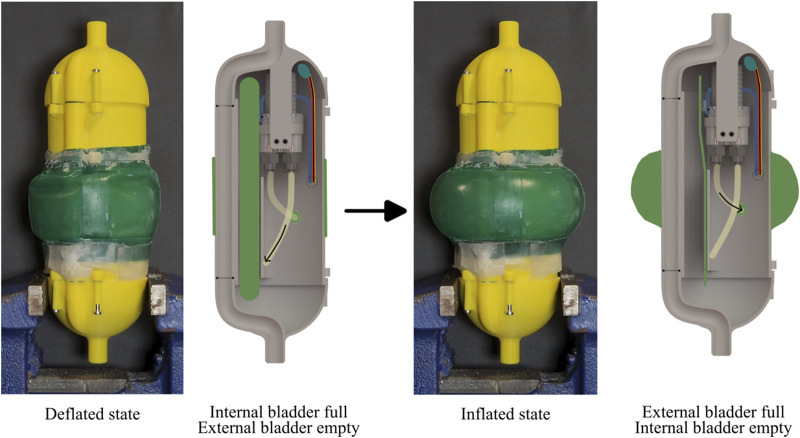
Water pump actuation of the shape change of the prototype robot. Left: Photograph and cross-sectional CAD schematic of the robot in the deflated state; the cross-section shows the full internal bladder. Right: Photograph and cross-sectional CAD schematic of the robot in the inflated state; the cross-section shows the empty internal bladder.

In addition, we also integrated the tethered water jet-based self-burrowing capability to this version of the robot. The water tether was initially attached to the top of the robot, and was detached when the robot had burrowed to its final depth of 9 inches (measured from between the top of the robot and the top surface of the sand). An internal channel in the 3D-printed body of the robot wrapped around the mechatronic components, directing the water flow to the nozzle at the bottom of the robot. A magnet attached to the end of the tether, along with a hall effect sensor within the robot, acted as a switch for the unburrowing mechanism; the unburrowing process began automatically when the robot had sensed that the tether was detached. The pump and the hall effect sensor were IP67 water-resistant and did not require waterproofing, but all other electronics were sealed within a TPU pouch (Figure 12).

#### 4.2.2 Operation and design limitations

**FIGURE 13:**
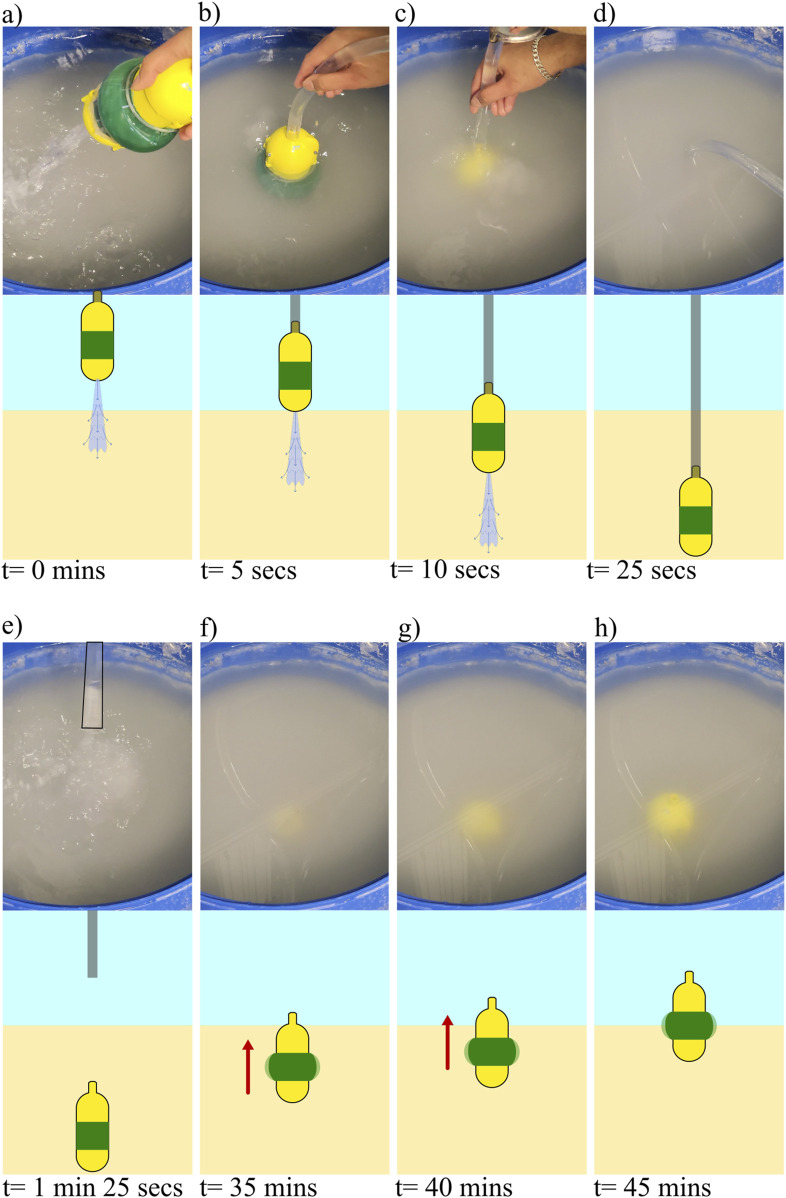
Demonstration of integrated system capable of water jet based burrowing, and untethered unburrowing via shape change. **(a–h)** Images from a video of the burrowing/unburrowing test (top) with corresponding schematics of the state of the robot in the GM (bottom) at the following steps of the process: **(a)** robot above the submerged GM with water flow turned on, **(b)** robot touching the submerged GM, **(c)** robot below the surface of the water and entering the surface of the GM, **(d)** robot at a depth of 230 mm below the surface of the GM, water flow is turned off, **(e)** tether pulled off after a rest period of 1 min, **(f)** the robot emerges above the surface of the GM after 33.5 min, **(g)** picture of the robot after 38.5 min, **(h)** Picture of the robot after reaching the top after 43.5 min.

In this system, the pump took 8 s to pump water from the internal bladder to the external bladder and another 8 s from the external bladder to the internal bladder. With the installed 1,300 mAh battery, the pump could operate around 50 min, which corresponded to 188 cycles with the cycle time of 16 s. This is three times as many cycles compared to the ${\text{CO}}_{2}$ canister approach. To test the system, we first attached the water tether to the top of the robot. During the burrowing process, we kept the external bladder at its minimum diameter for the minimal drag. We supplied water at a flow rate of 0.19 L per second to the other end of the tether using a diaphragm pump (Figure 13a). We then dropped the robot in the same submerged GM test setup as in previous tests Figure 13b, and the robot started burrowing in Figure 13c. Once the robot had reached a predetermined depth (230 mm), we turned off the water supply (Figure 13d). After waiting a minute for the fluidized sand to settle, we manually pulled the tether off the robot (Figure 13e). Qualitative visual inspection suggests that the robot did not rise in height during the tether removal (although this was not quantitatively measured). It is likely that because of the weight of the substrate above the robot, the robot stayed at that depth while the tether was detached. Once the tether was detached, the system automatically started the internal pump for the unburrowing mechanism. We observed that the robot first emerged on the surface at the 35 min mark (Figure 13f) and continue to burrow out further (Figure 13g). We observed that the robot was able to burrow out from a depth of 230 mm of submerged GM over 43 min (Figure 13h). A limitation of this hydraulic system approach is that because each cycle takes longer compared to that of the pneumatic system, the robot required more time to burrow out. This demonstration showed that the cyclic radial expansion of the bladders were indeed able to achieve untethered unburrowing of the robot out of submerged GM.

## 5 Conclusion

In this paper, we present a bio-inspired robot capable of both burrowing into, and without the aid of a tether, burrowing out out of submerged granular media (GM). The system uses a tethered water-jet-based approach for fluidization-based self-burrowing in submerged GM, and we have shown that the larger the flow rate of the jet, the greater the drag reduction and the faster the self-burrowing. For unburrowing, we demonstrate an untethered soft volume-change-based mechanism that uses periodic radial expansion to burrow out of both dry and submerged GM. We showed that for the unburrowing mechanism at a given activation pressure, the Advancement per cycle is inversely proportional to the depth within the GM, while the Slip per cycle does not reduce comparatively. In addition, we explored how the shape of the bladder and the weight of the system affects the burrowing-out speed by testing both the half-bladder and full-bladder variations with three different weights. We also optimized the unburrowing performance by reducing the exhaust cycle time. Lastly, we demonstrated a robot that achieved both burrowing and untethered unburrowing from a submerged GM, comparing two approaches of either a ${\text{CO}}_{2}$ canister-based pneumatic system or a pump-based hydraulic system, which proved that the system could autonomously unburrow from a depth of 230 mm of submerged GM over 43 min.

Potential applications of this work range from long-term environmental monitoring in sensitive marine ecosystems to underwater archaeology. Future work could investigate different bladder shapes to increase unburrowing performance in GM. In addition, the effects of varying nozzle diameters or the effects of having multiple nozzles on the burrowing performance could be studied. Also, onboard pumping methods for fluidization could be studied to make the system completely untethered.

## Data Availability

The raw data supporting the conclusions of this article will be made available by the authors, without undue reservation.
