# Supplementary material for: Burrowing and unburrowing in submerged granular media through fluidization and shape-change
Source: Front Robot AI. 2025 Jul 31;12:1546407. doi: 10.3389/frobt.2025.1546407 (PMC12351326; doi:10.3389/frobt.2025.1546407)
Supplement: Supplementary file 2 [file Supplementaryfile1.pdf]

Supplementary Material

1 COMPARISON OF WATER SUBMERGED AND DRY GRANULAR MEDIA FORCES

Below we provide justification for studying granular media penetration and drag force, and robot behavior, in dry granular media and comparing to we granular media. In short, 1) it is substantially easier to reset the compact ratio of dry GM, which leads to more consistent results, and 2) using pneumatic pressure for unburrowing in submerged GM causes the system to be buoyant compared to the surrounding medium, which might unwittingly aid the burrowing-out locomotion.

In submerged GM the influence of buoyancy and the water flow can cause different physics. We performed many experiments in dry sand because we have a very quick and reliable way of "resetting" the sand bed to an undisturbed state. So we can perform design studies relatively quickly. In submerged sand this is much more challenging to do because we do not have a water fluidized sand bed. The challenge with resetting water submerged sand is that it takes a long time for the sand to re-settle after it is fluidized. This complicates design approaches that rely on empirical testing.

However, unburrowing in dry sand is expected to be much more challenging than in fully submerged sand. This is because:

- The frictional forces in submerged sand are lower than dry sand since buoyancy effects reduce the sandgrain weight in water and thus reduce the contact normal and friction forces. We have performed our own experiments that show a 30% force reduction on a dragged object moving through submerged sand (see below).
- When a pneumatic air-bladder system is used, the added buoyancy effect from the inflated air bladder in water will only help unburrowing.

In the plot below we show a comparison of vertical insertion and horizontal drag of a small rectangular intruder in identical granular media, with and without water. The water case is fully submerged well above the surface of the sand. The comparison forces show a significant reduction in both the vertical penetration resistance force (green), and the horizontal drag resistance force (blue) on the intruder in water submerged sand. The force is reduced by approximately 30% in the drag direction, and 50% in the vertical penetration.

These results indicate that experiments in dry sand present a "worst case" scenario for resistive forces. We have added significant restructuring of the comments around wet/dry sand to the manuscript to reflect these important points you raise.

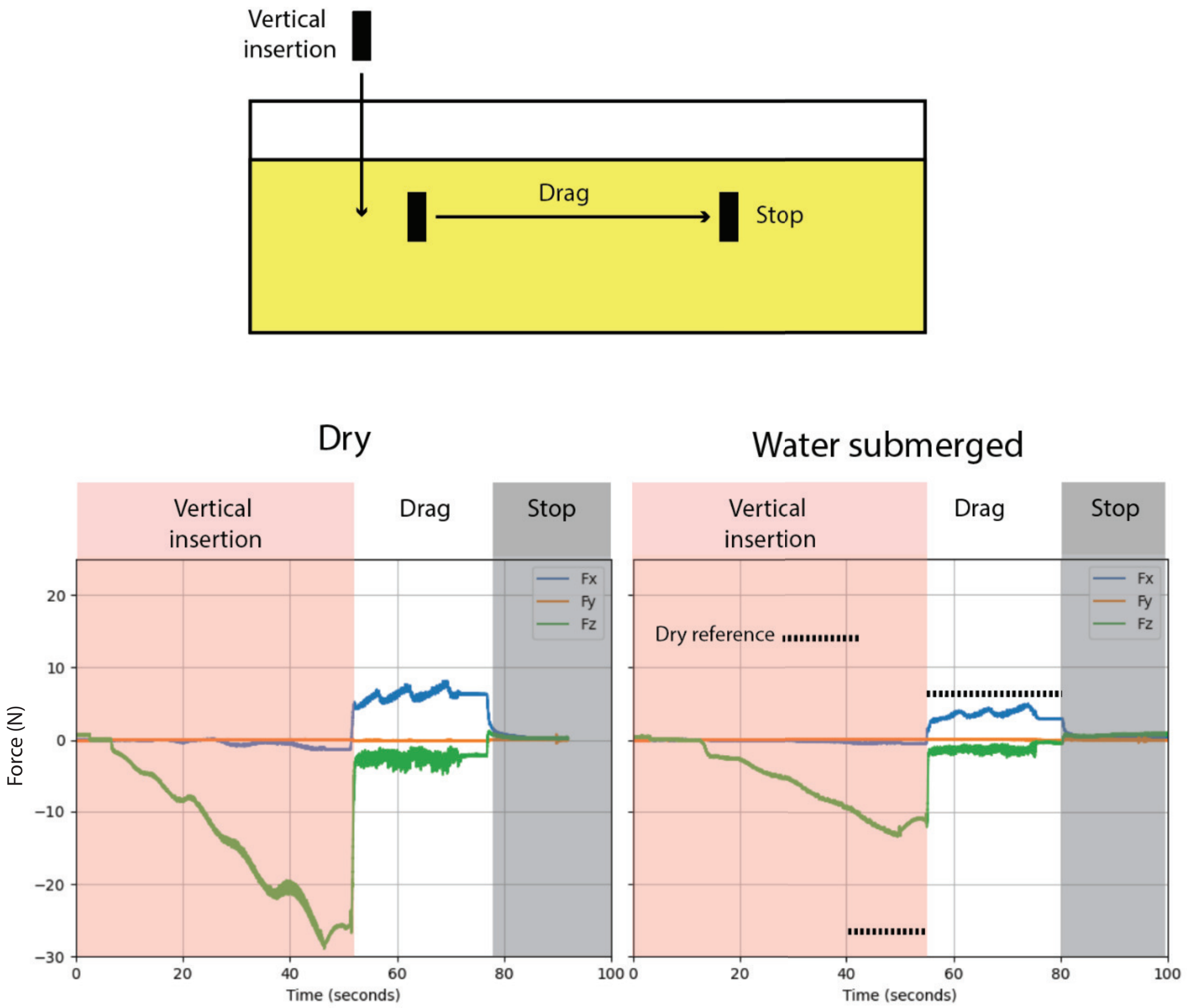

Figure S1. Comparison between drag force on a flat plate in granular media in air (dry; left), and water submerged granular media (right).
